# Curli mediate bacterial adhesion to fibronectin via tensile multiple bonds

**DOI:** 10.1038/srep33909

**Published:** 2016-09-22

**Authors:** Yoo Jin Oh, Michael Hubauer-Brenner, Hermann J. Gruber, Yidan Cui, Lukas Traxler, Christine Siligan, Sungsu Park, Peter Hinterdorfer

**Affiliations:** 1 Institute of Biophysics, Johannes Kepler University Linz, Gruberstrasse 40, 4020 Linz, Austria; 2 Center for Advanced Bioanalysis (CBL), Gruberstrasse 40, 4020 Linz, Austria; 3 Mechanobiology Institute, National University of Singapore, Singapore 117411, Singapore; 4 School of Mechanical Engineering, Sungkyunkwan University, Suwon 440746, Korea

## Abstract

Many enteric bacteria including pathogenic *Escherichia coli* and *Salmonella* strains produce curli fibers that bind to host surfaces, leading to bacterial internalization into host cells. By using a nanomechanical force-sensing approach, we obtained real-time information about the distribution of molecular bonds involved in the adhesion of curliated bacteria to fibronectin. We found that curliated *E. coli* and fibronectin formed dense quantized and multiple specific bonds with high tensile strength, resulting in tight bacterial binding. Nanomechanical recognition measurements revealed that approximately 10 bonds were disrupted either sequentially or simultaneously under force load. Thus the curli formation of bacterial surfaces leads to multi-bond structural components of fibrous nature, which may explain the strong mechanical binding of curliated bacteria to host cells and unveil the functions of these proteins in bacterial internalization and invasion.

Bacteria have developed a huge variety of sophisticated molecular strategies in order to colonize their hosts, to build up persistent infections, and to bypass the host’s defense mechanisms. One of these strategies is to use extracellular adhesion molecules which is often mediated via fibrous structures. These microbial filaments, e.g. curli^1^, flagella, and pili^2^ are key molecular players in the bacterial adhesion and initiate aggregation of bacterial cells to promote the formation of highly resistant and impervious biofilm. Among them, curli fibres exhibit typical characteristics of amyloids and their biogenesis and amyloid fibre formation. Microbial curli from many Enterobacteriaceae and other fungal amyloid domains from *Candida albicans*^2^,^3^ and yeast cells^4^ are well-known for being responsible for several neurodegenerative diseases like Alzheimer’s, Parkinson’s, and autoimmune disease^5^,^6^,^7^. Similarly, microbial amyloid curli are important molecular players in the adhesion to the host’s surface fibres^8^, such as fibronectin^9^,^10^,^11^, a large and essential cellular multi-domain glycoprotein with multiple adhesive properties. Despite structural^12^ and nano-mechanical^13^,^14^ studies, the exact mechanism of binding and the molecular nature of the specificity remain obscure.

Investigating bacterial surfaces at the single molecular level requires high resolution microscopy methods. Atomic force microscopy (AFM) is an appropriate tool for investigating cellular and microbial surface nanostructures in native environments^15^,^16^,^17^. An exquisite feature of AFM is the potential to explore molecular forces with high precision and accuracy from interactions of single molecular receptor/ligand pairs under physiological conditions^18^,^19^,^20^. From such single molecular force spectroscopy (SMFS) experiments, structural insight into binding pockets, chemical rate constants, and interaction energy landscapes can be deduced. Thus, we exploited SMFS to reveal essential details about how curli binds to fibronectin (FN). In particular, we used curli both in its monomeric isolated form and in its oligomeric fibre state expressed on bacterial surfaces to study its binding forces to various fibronectin constructs. We initially measured specific interaction forces of bacteria curli protein CsgA monomers to multi-domain full-length fibronectin (FN), isolated domain III (FN III), or a peptide with the core RGD sequence (RGD). Dimeric FN consists of two FNIII domains, each of which contains one wide-spread and specific binding sequence RGD (Fig. S1).

AFM cantilever tips functionalized with the FN constructs were repeatedly brought into contact with surfaces coated with CsgA to allow molecular bond formation (Fig. 1A–C). The cantilever was then retracted (grey arrows in the sketches of Fig. 1A–C) at a defined distance and speed. During the retraction, an increasing force load was applied to the molecular bond that was directly measured from the downward deflection of the AFM cantilever (red arrows in Fig. 1A–C). Finally bond breakage occurred at a critical unbinding force (circles in Fig. 1A–C). In most of the force-distance curves recorded with RGD (Fig. 1A), FN III (Fig. 1B), and FN (Fig. 1C) single-molecule unbinding signatures were observed. Multiple unbinding events were occasionally detected for FN as a result of several sequential FN/CsgA bond ruptures.

The collected unbinding forces were gathered, summed up, and normalized to calculate the empirical probability density functions (PDFs) (Fig. 1D). PDFs contain the original data and can be viewed as the equivalent of continuous force histograms. As such, they show the unprocessed distribution of unbinding forces. Their maxima represent the most probable measured unbinding force values and the uncertainties (widths) reflect the stochastic nature of the unbinding process rather than the experimental error. The values for the maxima were remarkably similar (Fig. 1D) for RGD (51 ± 19 pN), FN III (43 ± 16 pN), and FN (57 ± 23 pN) (P = 0.77 for RGD/FN III, P = 0.29 for RGD/FN, P = 0.69 for FN III/FN), suggesting that binding to CsgA might occur through the same binding epitope. Binding was also specific, as verified in blocking experiments by adding the RGD peptide into solution (Fig. 1E). This is strong evidence that the binding of all three FN constructs to the CsgA monomer is RGD-dependent.

We characterized the molecular bonds further to attain the kinetic off-rate constant K_off_ and the length scale of the interaction energy potential X_β_^21^,^22^. K_off_ and X_β_ characterize the molecular transition during dissociation (Fig. 2A). We varied the pulling rate in our SMFS experiments and plotted each measured unbinding force as a function of its loading rate (Fig. 2C–E). In accord with the model of Bell and Evans^21^,^22^, we observed a linear rise in the unbinding force with respect to a logarithmically increasing loading rate (Fig. 2B–E). K_off_ and X_β_ were evaluated from data fits (Fig. 2): K_off_ = 1.19 ± 0.08 s^−1^ and X_β_ = 2.84 ± 0.05 Å for RGD/CsgA, K_off_ = 1.2 ± 0.11 s^−1^ and X_β_ = 4.04 ± 0.08 Å for FN III/CsgA, and K_off_ = 1.12 ± 0.06 s^−1^ and X_β_ = 3.67 ± 0.05 Å for FN/CsgA binding. K_off_ was similar for all three interactions (P = 0.74 for RGD/FN III, P = 0.6 for RGD/FN, P = 0.45 for FN III/FN), whereas X_β_ varied to some extent (P = 0.26 for RGD/FN III, P < 0.001 for RGD/FN, P < 0.001 for FN III/FN). The good agreement among the K_off_ values verified the RGD epitope’s almost equal bond strength when RGD monomer, FN III, and FN bind to CsgA. Differences in X_β_, which represents an estimate of the bond length during the dissociation process, may be explained by the variable spatial accessibility of the RGD binding sites and the different lengths of the FN constructs. Bond lifetimes τ directly calculated from the kinetic off-rates according to τ = K_off_^−1^ revealed a relatively short bond survival of about 0.85 s.

We extended our SMFS studies to directly measure the interaction forces of the FN constructs with fibrous curli expressed on intact living bacterial surfaces and placed *Escherichia coli* (*E. coli*) onto gelatin-coated mica surfaces^23^. Expression of curli amyloids on the surface of *E. coli* is enhanced during growth on a solid medium^24^ and with the invasion of eukaryotic cells^8^. We have previously shown that different stages of curliation can be mimicked by using different mutants, i.e. wild type (WT), CsgA knock-out mutant (CsgA(−)), and CsgA-over-expressing mutant strains (CsgA(+))^24^. AFM images were acquired in liquid to resolve curli production from the bacterial surface topology that resulted from the formation of curli complexes on the bacterial surface of CsgA(+) (Fig. 3C) after induction of curli expression, whereas the mutant lacking curli expression showed a smoother surface structure (Fig. 3B). Molecular interactions to bacterial surfaces were studied using AFM tips conjugated with RGD, FN III, and FN (Fig. 3A). It is important to note that, in contrast to the monomeric CsgA surfaces, multiple-bond rupture events with wide rupture lengths were observed here (Fig. S2). CsgA(+) and WT showed high binding probabilities in their interactions with RGD, FN III, and FN (9–15%). In contrast, CsgA(−) devoid of the CsgA protein on its bacterial membrane showed a very low binding probability (1–3%). For WT and CsgA(+), the unbinding forces that originated from single-bond breakages with RGD, FN III, FN mostly fell in a force window between 45–60 pN (Fig. 3D). This compares nicely with the forces observed for monomeric CsgA and implies that the interaction between RGD and CsgA drives bacterial adhesion when curli fimbriae and fibronectin are involved.

The work required to de-adhere molecular complexes is a quantitative measure for molecular adhesion strength. Following this conception, we identified the adhesive interaction strength of RGD, FN III, and FN to curliated bacteria (CsgA(+)) by determining the work done by the pulling cantilever to detach the FN constructs from the bacterial surface. This non-equilibrium work for breaking the overall adhesion was calculated from the cumulative path integral of unbinding in force-distance curves (Fig. S2)^25^. It includes contributions from deforming the bacterial membrane and from extending the curli fibers involved in molecular complexation, as well as the energy required for breaking all molecular connections (Fig. S2). Histograms of the calculated de-adhesion work arising from the unbinding of RGD and FN III displayed characteristic maxima (Fig. 3E) that were similarly distributed and consisted of three and four individual peaks of quantized nature, respectively. This implies that up to four tip-adorned molecules could access the bacterial membrane to contribute to the overall adhesion process. A work quantum of ~570 pN·nm was spent when only one molecular bond was involved in adhesion. For several molecular connections (n > 1), the work per bond was slightly lower and amounted to ~430 pN·nm (cf. Fig. 3E). This decrease in work consumption per bond might indicate that the energy for membrane deformation was partially shared among the bonds, as expected from the parallel bond detachment observed. In contrast to RGD and FN III, the fully extended wild type FN showed a broad work distribution lacking resolution of individual bonds with the most probable value being about seven- to eight-fold the work quantum required for single RGD de-adhesion.

We then studied the adhesion strength of single bacteria with fibronectin in microbial cell force spectroscopy experiments using bacterial cell probes (Fig. 4A)^26^,^27^,^28^. Bacterial cells (WT, CsgA(−), CsgA(+)) were immobilized onto a tip-less cantilever and interaction forces between the bacteria and the fibronectin coated surface (Fig. 4A) were followed in force-distance curves (Fig. 4B). Most of the force curves (binding probability 94%) recorded using CsgA(+) cell probes expressing curli showed multiple force spikes with a final large unbinding event (Fig. 4B) of 413 ± 102 pN (Fig. 4C) at full bacterial de-adhesion. De-adhesion occurred at extended unbinding lengths of 568 ± 152 nm (Fig. 4C, inset), reflecting the tensile nature of the binding complex. The last unbinding event comes from simultaneous multi-bond breakage, whereas the preceding force spikes (white arrow) are indicative of several gradual step-by-step ruptures that may arise from sequential molecular unbinding events or tether-like structures^16^,^17^. The resulting de-adhesion rupture work, calculated as described above, was broadly distributed around 10950 ± 2710 pN·nm (Fig. 4D). In contrast, WT and CsgA(−) (Fig. 4B,C) cell probes detected lower forces (55 ± 29 pN and 113 ± 58 pN, respectively) and shorter unbinding lengths (310 ± 207 nm for WT and 47 ± 28 nm for CsgA(−), Fig. 4C, inset). WT de-adhesion rupture work (Fig. 4D) originated from specific binding (binding probability 76%) and its magnitude of 643 ± 544 pN·nm is in line with the breakage of a few individual bonds. CsgA(−) adhered nonspecifically (binding probability 22%) and the adhesion (de-adhesion work of 1377 ± 488 pN·nm, Fig. 4D) most likely arose from long flagella produced on the bacterial surface^24^. WT produces much less flagella^24^, so that specific binding is not expected to be impaired.

*E. coli* cells producing CsgA (CsgA(+) mutant) bound to surfaces of the extracellular matrix protein FN through specific multiple molecular connections via the RGD binding motif. Several pilus-associated adhesin also interact specifically with other ECM protein in a similar force range (75 ± 28 pN for interactions between pilin subunit SpaC and collagen^29^). The kinetic off rates, however, varied to some extend due to the different nature of the adhesion mechanisms^29^. In addition, the de-adhesion force of CsgA(+) from the FN surface (413 ± 102 pN) was comparable to single *Streptococcus mutants*-Salivary agglutinin interaction (~500 pN, when about 10 adhesins are involved in)^27^.

In a simple bond analysis model it was shown that the simultaneous breakage of *N* number of bonds occurs at a force less equal than *N*-times the force for breaking a single bond^30^. Thus, the ratio of the force required to dissociate CsgA(+) from an FN surface to the unbinding force of a single CsgA/RGD bond, 413 pN/51 pN = 8.1, implied that at least 9 bonds were broken simultaneously. In addition, several bonds may have disrupted sequentially (Fig. 4B) before the final unbinding occurred. This results in a total of around 10 specific bonds and a bond density of about 100/μm^2^ (the cell contact area of 116520 nm^2^ was approximated via the Hertz model^26^,^31^). This value appears reasonable, as similar values have also been found in other bacterial systems^27^,^32^,^33^. For a single RGD/CsgA bond, we attained a short lifetime τ = 1/K_off_ of about 0.85 s. The lifetime of 10 such bonds lies between 2.9 and 8.5 s (simultaneously *vs*. sequential unbinding^30^), which does not mirror long-lasting interactions. However these estimates do not consider rebinding effects that are well expected when two fibrillary proteins, FN and CsgA curli, are involved in the binding process. In addition, the overall work for de-adhering curliated bacteria from the FN surface under mechanical force does not only contain contributions from the binding energy of the molecular bonds, but also from stretching the fibrous proteins and deforming the bacterial membrane. We found that the work required to finally dissociate CsgA(+) from the FN surface amounted to a value as high as 11000 pN·nm, which equals 2750 times the thermal energy *k*_*B*_*T*. This value can be taken as the upper threshold that allows these bacterial cells to resist detachment from external forces induced by external fluid flows.

Bacteria follow diversified strategies to accomplish attachment and internalization into hosts. For example, Type IV pili in *Neisseria gonorrhoeae* generate a high motile force to swim close to host cells and also invade host cells through binding to receptors. Since Type IV pili generate high contractile forces^16^, several pilus-host receptor binding events are sufficient to induce bacterial internalization^34^,^35^. On the contrary, curli in *E. coli*, which are expressed by many pathogenic isolates of *E. coli* and present on several *Salmonella* strains^6^,^8^, do not generate motile force and are mainly used to bind fibronectin^8^. We have shown that curliated *E. coli* form quantized and multiple bonds of high tensile strength with fibronectin through specific RGD/CsgA connections that lead to quasi-irreversible bacterial attachment. The suchlike accomplished tight binding may allow bacteria to resist detachment from host cells induced by shear force in blood and interstitial fluids to facilitate bacterial internalization and invasion. Our insights provided by single molecule and microbial cell force spectroscopy measurements constitute the basis for unraveling novel mechanisms that govern bacteria-host cell interaction. This also offers exciting perspectives for controlling bacteria-host binding and thus opens new possibilities for alternative therapeutic strategies.

## Methods

### Bacteria strain, mutants, and cultivation

*E.coli* K-12 strain W3110 (wild-type (WT) strain), its *CsgA* single-gene knock out mutant (CsgA(−)), and *CsgA* overexpressing mutant (CsgA(+)) were obtained from National Bio Resource Project (NBRP, Japan). For maintaining the plasmid encoding *CsgA* in CsgA(+) mutant, choloramphenicol (Sigma Chemical Co, St. Luis, MO) was used at a final concentration of 30 μg/ml during cell culture^36^. *E. coli* strains were grown overnight in 15 ml round poly propylene tubes containing Luria-Bertani medium (LB) (Difco) at 37 °C and 220 rpm. The cultures were then diluted 100-fold in fresh LB medium and continuously grown at 37 °C with aeration until their optical density at 600 nm reached 0.4. CsgA(+) mutant was grown for an additional 2 h after adding 1 mM isopropyl-β-D-thiogalactoside (IPTG, Sigma) to overproduce CsgA^24^. CsgA monomers were purified as published^36^.

### AFM tip amino-functionalization

Commercially available AFM cantilevers (MSCT, Bruker, CA, USA) with a nominal spring constant of 0.01–0.03 N/m were functionalized with amino groups by using a 3-aminopropyltriethoxysilane (APTES) coating procedure^37^. All steps of tip functionalization were performed according to the instructions found at http://www.jku.at/biophysics/content/e257042.

### Conjugation of fibronectin through lysine residues to the cantilever

A heterobifunctional poly(ethylene glycol) (PEG) linker with a length of 6–9 nm as synthesized in our lab was attached to the 3-aminoprobyl triethoxysilane (APTES)-coated cantilever via its N-hydroxysuccinimide (NHS) ester group and the aldehyde function on the free-tangling end of PEG was used for coupling or protein via one of the lysine residues. The bond was fixed by reduction with NaCNBH_3_. The overall procedure was done as described before^37^. Human fibronectin (Yo Proteins AB. Huddinge, Sweden) in phosphate-buffer saline (PBS, pH7.4) at 0.2 mg/ml (final conc.) was used for tip functionalization. The functionalized cantilevers were washed with PBS and stored in PBS at 4 °C.

### Conjugation of recombinant human fibronectin fragment 3, and CsgA monomers through histidine residues of His_6_-tagged protein

The maleimide-PEG linker was attached to the APTES-coated cantilever by incubating the cantilevers for 2 h in 500 μl of chloroform containing 1 mg of maleimide-PEG-NHS (Polypure, Oslo, Norway) and 30 μl of triethylamine. Subsequently, cantilevers were washed with chloroform and dried with nitrogen gas. 50 μl of 4 mM thiol-trisNTA in Hepes-EDTA buffer was first mixed with 3 μl of 100 mM tris(carboxyethyl)phosphine (TCEP) hydrochloride and then with 3 μl of nominally 1 M Hepes (prepared from 1 M Hepes acid solution by adjusting pH 10.0 with 20% NaOH). The cantilevers were immersed in this solution for 2 h and then washed with Hepes buffer. Subsequently, the cantilevers were incubated for 5 min in 50 μl of Hepes-buffer saline (HBS) containing 2 μl of 5 mM NiCl_2_ to obtain a final concentration of 200 μM NiCl_2_. After Ni^2+^ loading, the cantilevers were incubated for 3 hours in a mixture of 2 μl of 5 mM NiCl_2_ and 50 μl of HBS containing 0.2 mg/ml of His_6_-tagged fibronectin fragment 3 ((FNIII), R&D systems, UK) or isolated CsgA-His_6_ monomer. After washing three times with HBS, the cantilevers were stored in HBS at 4 °C.

### Conjugation of RGD through cysteine

The maleimide-PEG linker was attached to the APTES-coated cantilever that was prepared as described above. First, 3 μl 100 mM TCEP was added to 50 μl of 4 mM RGD peptide in PBS, before 3 μl of 1 M Hepes (pH10.0) was added. The cantilevers were incubated in this solution for 2 h and then washed with PBS three times. The sequence of the RGD peptide studied in this paper was CGGRGDS (custom-synthesized by Peptide 2.0, VA, USA).

### Sample preparation

For single molecular force spectroscopy (SMFS) measurements on intact bacterial surfaces, bacteria were immobilized on gelatin-coated mica surface as described before^23^. A gelatin solution was prepared by dissolving 500 mg gelatin (Gelatin from porcine skin, G6144, Sigma) in 100 ml of distilled water at 60 °C. After cooling to 40 °C, both sides of freshly cleaved mica surfaces were vertically dipped into the gelatin solution and allowed to air dry by standing on a paper towel. Bacterial suspensions were centrifuged (1500 × g) for 2 min and the pellet was re-suspended in PBS buffer. This washing step was repeated two times. The final bacterial collection was then re-suspended in 100 μl of PBS buffer and this aliquot was pipetted onto the gelatin-coated mica surface. The samples were allowed to stand for 30 min, and then rinsed with 10-fold diluted PBS buffer. This method for sample preparation did not involve chemical treatment or a drying process.

### Immobilization of bacteria on tipless cantilever

Commercially available tipless cantilevers (MLCT, Bruker, CA, USA) with a nominal spring constant of 0.03–0.1 N/m were functionalized with poly-L-lysine and glutaraldehyde to immobilize bacteria onto the cantilever. The cantilever was dipped in a 0.01% concentration of a 150–300 kDa poly-L-lysine (P4832, Sigma-Aldrich, USA) solution and dried at room temperature. Subsequently, the cantilever was immersed in 0.1% glutaraldehyde (Sigma-Aldrich, USA) solution for 15 min. After incubation, the tipless cantilever was washed with deionized water more than 10 times. The 100 μl bacteria suspension was applied to the tipless cantilever and incubated for 1 h. With this procedure, the cantilevers were sparsely covered with bacteria, i.e. within a surface concentration of ~5–10 cells/100 μm^2 ^38. Therefore, with the given tilting angle of the cantilever during experiments (~20°), the probability is very low that two or more bacterial cells touch the surface at the same time with the applied moderate forces (400 pN).

### Single molecular force spectroscopy measurements

Force distance measurements were performed at room temperature (~25 °C) using tips of 0.01–0.03 N/m nominal spring constants, conjugated with fibronectin constructs (FN, FN III, RGD). Spring constants (*K*_*C*_) precisely of AFM cantilever were determined by measuring the thermally driven mean-square bending of the cantilever using the equipartition theorem in an ambient environment^39^. The deflection sensitivity was calculated from the slope of the force-distance curves recorded on bare silicon substrate. Determined spring constants ranged from 0.009 to 0.012 N/m.

In the force spectroscopy measurements, force distance curves were acquired by recording at least thousand force distance cycles with vertical sweep rates between 0.5 and 10 Hz at a z-range of typically 500–1000 nm, resulting in loading rates from 100 to 5000 pN/s. The loading rates were determined by multiplying the pulling velocity with the effective spring constants (*K*_*eff*_), which were obtained by the spring constant of the cantilever (*K*_*C*_) and the spring constant of the PEG-linker (*K*_*L*_), according to *K*_*eff*_ = [1/*K*_*C*_ + 1/*K*_*L*_]^−1^. *K*_*L*_ was calculated by fitting the force distance curves with the worm-like chain model.

The relationship between experimentally measured unbinding forces and parameters from the interaction potential were taken from the kinetic models of Bell^21^, and Evans and Ritchie^22^. Here, the unbinding force F^*^ as a function of the loading rate *r* is described by F^*^ = (*k*_*B*_*T*/X_β_)· ln(r·X_β_/K_off_·*k*_*B*_*T*), where *k*_*B*_*T* is the Boltzmann thermal energy, X_β_ marks the thermally averaged projection of the transition state along the direction of the force, and K_off_ is the kinetic off rate for the bond in an absence of applied load^39^.

### Calculation of de-adhesion work from force-distance curves

**Figure 3:**
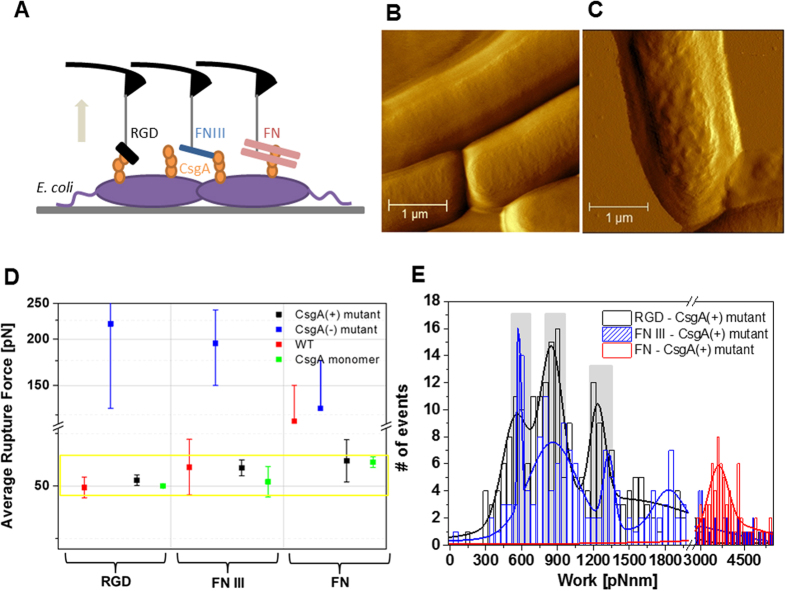
Single-molecular force spectroscopy experiments on *E. coli* bacteria. (**A**) AFM tips contained RGD, FN III, FN; surface-bound bacteria were WT, CsgA(−), and CsgA(+). (**B**) AFM amplitude images of CsgA(+) mutant without curli expression, and (**C**) CsgA(+) mutant after curli expression has been induced. (Scale bar = 1 μm). (**D**) Single-molecular unbinding forces measured at a pulling speed of 500 nm/s. (RGD, n = 3000; FN III, n = 2000; FN, n = 2000; for each bacterial mutant using 2–3 different cantilevers and bacterial surfaces from 2–3 different batches) Force values from CsgA(−), WT, and CsgA were mainly collected within the yellow box indicated by a force range of 45–65 pN. The small number of detected forces on CsgA(−) (blue dots) showed irregular distributions indicative of multiple non-specific interactions that most likely arose from the production of flagella in this mutant^24^. (**E**) Histogram of de-adhesion work for the dissociation of RGD, FN III, FN from the surface of CsgA(+) at a pulling velocity of 1000 nm/s. Each distribution contains calculated adhesion work from 1000 force curves. The most probable de-adhesion work values from the maxima were fitted with multi-Gaussian distributions. Grey bars indicate work quanta. Single RGD required a de-adhesion work of 552 ± 17 pN·nm, the second and third peak were at 840 ± 10 pN·nm, and 1234 ± 8 pN·nm. FN III showed maxima at 580 ± 1, 856 ± 11, and 1326 ± 6. FN required much higher de-adhesion work and the most probable value was 3610 ± 30 pN·nm.

**Figure 4:**
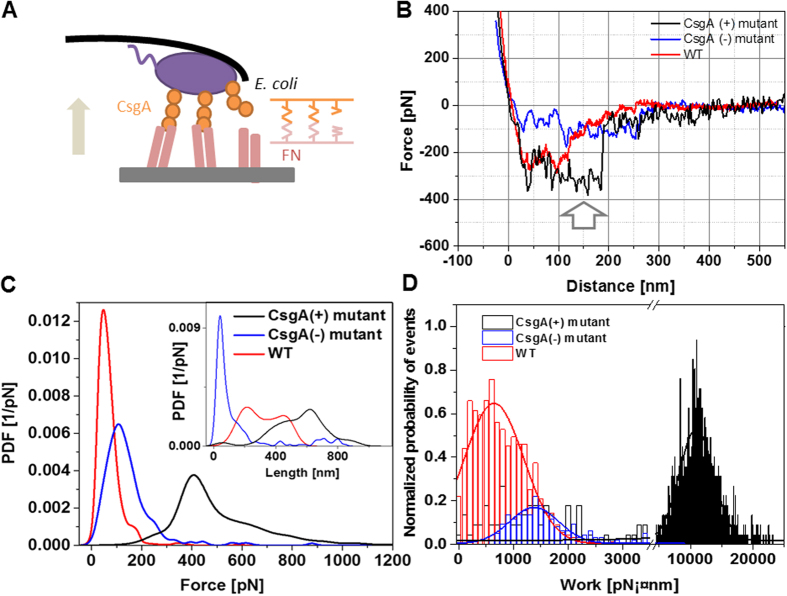
Microbial cell force spectroscopy experiments on FN-coated surfaces. (**A**) AFM tip-less cantilevers containing CsgA(+), CsgA(−), and WT. Surfaces were coated with FN. (**B**) Typical force-distance curves measured between bacterial mutants (WT, CsgA(−), CsgA(+)) and FN on the surface. (**C**) PDFs of unbinding forces and unbinding lengths (inset) (n = 1000 for each). (**D**) Histogram of de-adhesion work upon dissociation of bacterial mutant (WT, CsgA(−), CsgA(+)) from the FN surface (n = 1000 for each mutant). The y-axis was normalized with respect to unbinding probability.

The work required for breaking the adhesion (de-adhesion work *W*) was calculated from the cumulative path integral of unbinding events in force-distance curves, according to *W* = ∫_c_ *F* · dx, where x is the pulling coordinate (molecular extension), and *F* is the force obtained from the deflection of the cantilever during retraction. Calculated de-adhesion works from individually measured force-distance curves were plotted in histograms and fitted with multi-Gaussian functions as shown in Figs 3E and 4D. 1000 force-distance curves from at least 5 different samples were taken to obtain the de-adhesion work distributions.

## Additional Information

**How to cite this article**: Oh, Y. J. *et al*. Curli mediate bacterial adhesion to fibronectin via tensile multiple bonds. *Sci. Rep.* **6**, 33909; doi: 10.1038/srep33909 (2016).

## Supplementary Material

Supplementary Information

## Figures and Tables

**Figure 1:**
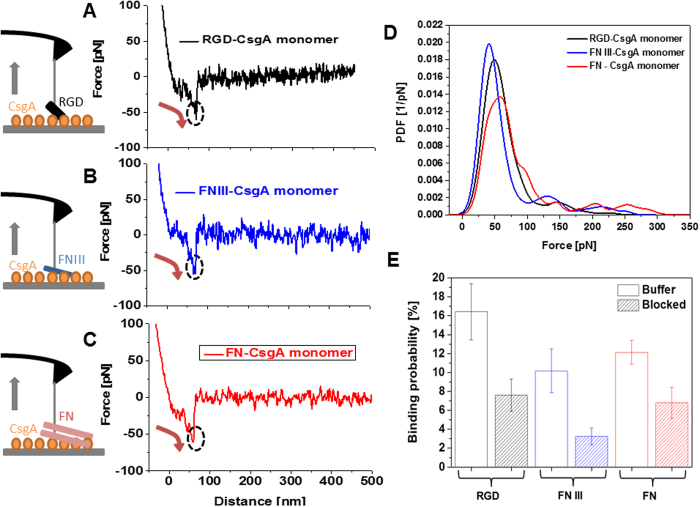
Single-molecular force spectroscopy experiments on surface-bound CsgA monomers. Typical force-distance curves recorded using AFM cantilever tips functionalized with (**A**) RGD. (**B**) FN III. (**C**), FN. Monomeric CsgA was tethered to silicon chip surfaces (sketches in (**A**–**C**)) via a flexible poly(ethylene glycol) (PEG) chain. Likewise, the RGD peptide, FN III, or FN were flexibly linked to AFM tips. The PEG linker that connected the molecules to the AFM tips and probe surfaces (Fig. S1) ensured sufficient motional freedom for unconstrained interaction measurements. Sketches on the left side show bond formation and the downward deflection of the AFM cantilever during retraction. Red arrows in force-distance curves indicate a bond load increase, circles mark bond rupture. (**D**) PDFs of unbinding forces at a retraction velocity of 500 nm/s. For each PDF 1000 force curve measurements were recorded. (**E**) Binding probability (defined as the percentage of force experiments displaying unbinding events) for RGD (n = 3000, 3 different tips), FN III (n = 3000, 3 different tips), and FN. (n = 6000, 6 different tips) Addition of RGD peptides into the measurement solution (blocked) resulted in a significant drop of the binding probability: from 16 to 7% for RGD (n = 3000, 3 tips), from 10 to 3% for FN III (n = 3000, 3 tips), and from 12 to 6% for FN (n = 3000, 3 tips).

**Figure 2:**
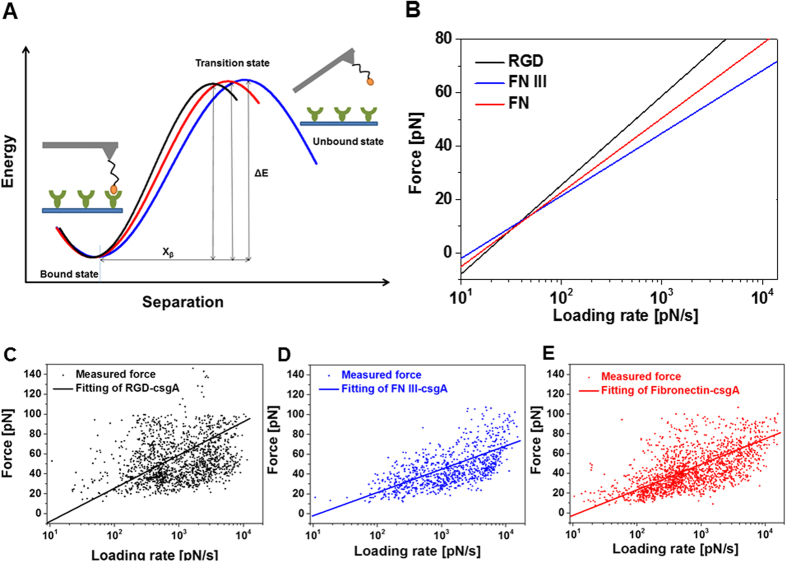
Interaction energy mapping by use of single-molecular force spectroscopy. (**A**) Scheme depicting the transition from the bound to the unbound state for the dissociation of RGD, FN III, and FN from CsgA. (**B**) Plot of unbinding force versus loading rate for AFM tips containing RGD, FN III, and FN dissociating from a CsgA-coated surface. Unbinding forces versus loading rate for tip-coupled (**C**) RGD, (**D**) FN III, and (**E**) FN. 5 different loading rates with 1000 force curves respectively were recorded and tested with at least 3 different cantilevers and surface preparations. CsgA monomers were bound onto silicon surfaces. The unbinding force F_i_ and effective spring constant (slope at rupture) were determined from force curves showing unbinding events. The loading rate r_i_ of every individual curve was calculated by multiplying the effective spring constant with the pulling speed. The data variance reflects the stochastic nature of the unbinding process and not the measurement error. Data were fitted using a maximum likelihood approach^40^. For this, the negative log likelihood *nll* was minimized by varying K_off_ and X_β_: *nll* = −Σ_i_ *p*(K_off_, X_β_; F_i_,r_i_), with *p* being defined in Evan’s single-energy barrier model^21^,^22^.
