# Supplementary material for: Curli mediate bacterial adhesion to fibronectin via tensile multiple bonds
Source: Sci Rep. 2016 Sep 22;6:33909. doi: 10.1038/srep33909 (PMC5031991; doi:10.1038/srep33909)
Supplement: Supplementary Information [file srep33909-s1.pdf]

SUPPLEMENTARY INFORMATION

Curli mediate bacterial adhesion to fibronectin via tensile multiple bonds

Yoo Jin Oh[1*], Michael Hubauer-Brenner[1,2], Hermann J. Gruber[1], Yidan Cui[3], Lukas Traxler[1], Christine Siligan[1], Sungsu Park[3,4], and Peter Hinterdorfer[1,2*]

[1]Institute of Biophysics, Johannes Kepler University Linz, Gruberstrasse 40, 4020 Linz, Austria

[2]Center for Advanced Bioanalysis (CBL), Gruberstrasse 40, 4020 Linz, Austria

[3]Mechanobiology Institute, National University of Singapore, Singapore 117411, Singapore

[4]School of Mechanical Engineering, Sungkyunkwan University, Suwon 440746, Korea

[*]Correspondence to yoo_jin.oh@jku.at, peter.hinterdorfer@jku.at

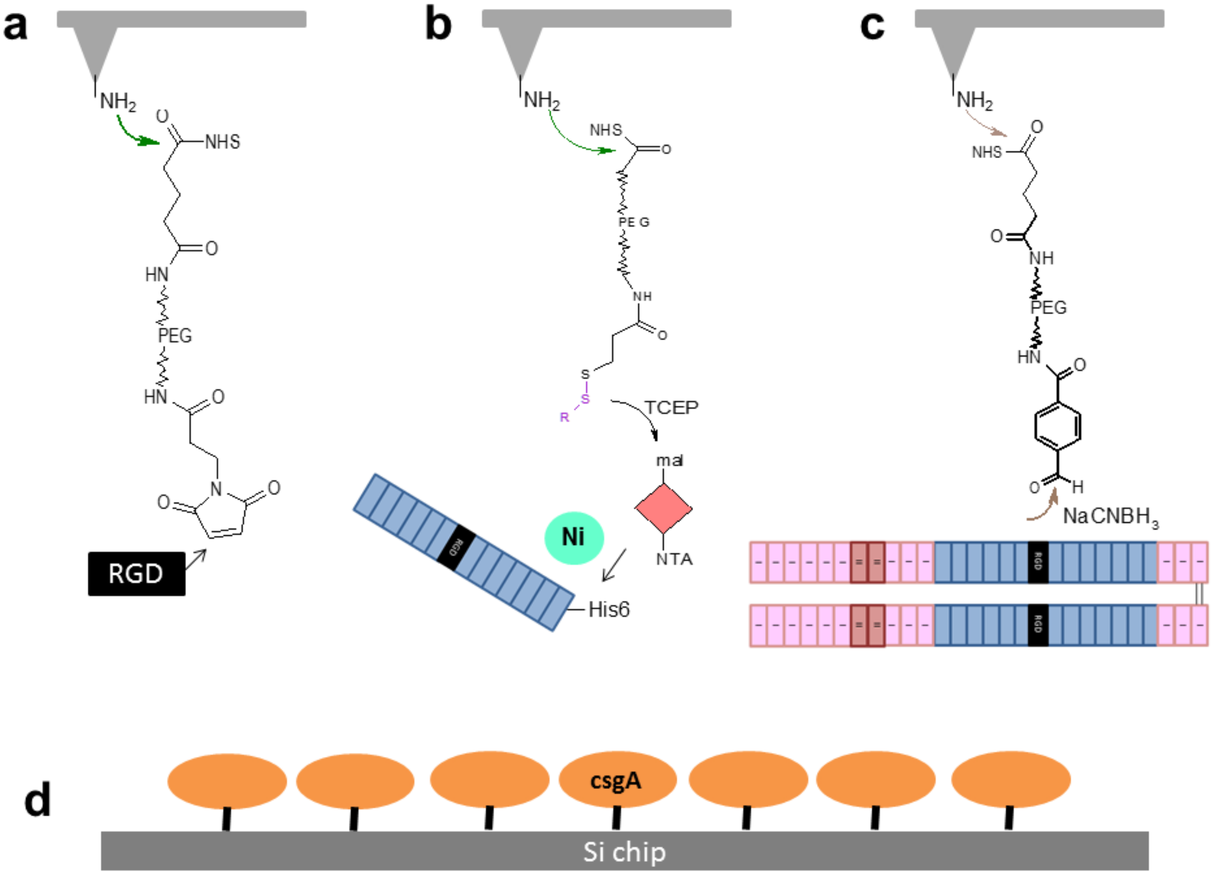

Figure S1| Scheme of immobilization strategy for single molecular force spectroscopy experiments. Tip and probe surfaces were amino-functionalized as a first step, before a flexible poly(ethylene glycol) (PEG) linker was added. In the final coupling step, proteins were bound to different end groups of the PEG linker. **a**, RGD peptide was bound to maleimide-PEF via cysteine. **b**, Fibronectin domain III (FN III) to tris-nitrilotriacetic acid (NTA)-PEG via C-terminal his_6 tag. **c**, Fibronectin (FN) to aldehyde-PEG linker via lysine residues. **d**, CsgA monomers to NTA-PEG via his_6 tag. For all fibronectin constructs (a,b,c) RGD epitopes are marked in black.

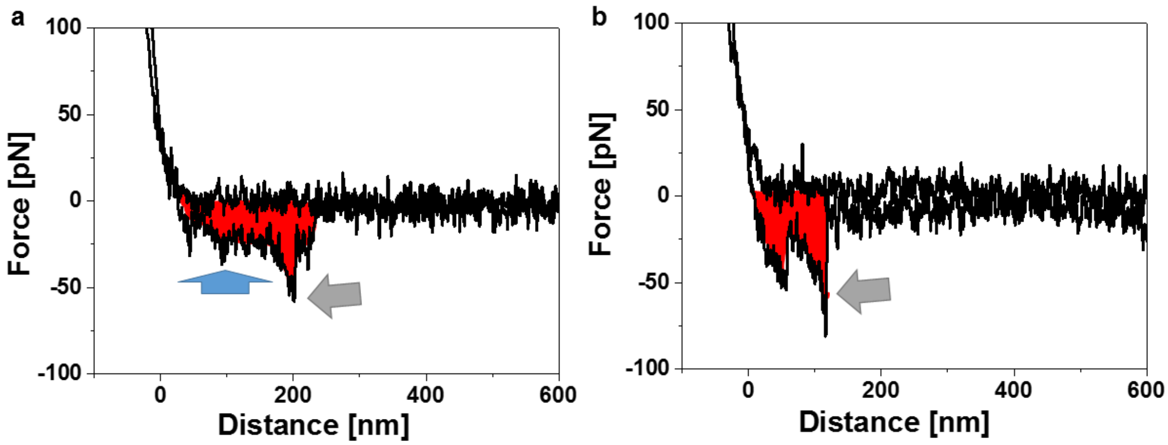

Figure S2| Typical force-distance curves recoreded from RGD/CsgA(+) interactions. Red area indicates the calculated de-adhesion work. **a**, Blue arrow shows membrane and curli fiber extension during retraction of the RGD-bound AFM tip. **b**, The grey arrow indicates a typical unbinding event between RGD and the CsgA(+) bacterial surface.
